# A wearable soft robot that can alleviate the pain and fear of the wearer

**DOI:** 10.1038/s41598-022-21183-7

**Published:** 2022-10-17

**Authors:** Youchan Yim, Yohei Noguchi, Fumihide Tanaka

**Affiliations:** University of Tsukuba, Tsukuba, Japan

**Keywords:** Information technology, Computer science

## Abstract

Social soft robotics may provide a new solution for alleviating human pain and fear. Here, we introduce a hand-held soft robot that can be clenched by the wearer. The robot comprises small airbags that can be inflated to provide the wearer with a feeling of being clenched. We then conducted an in-depth study of 66 adults who participated in a pain research protocol using thermal stimulation to investigate the effect of wearing the robot on pain perception and fear of injections. Pain assessment scale scores for perceived pain decreased significantly $(p < 0.05)$ when participants wore the robot compared with the baseline condition in which the robot was not worn. In addition, the saliva test results showed a downward trend in oxytocin level when the robot provided the wearer with haptic feedback via the inflation of the internal airbags in response to the wearer’s clench. Furthermore, the negative psychological state of participants, as measured using the positive and negative affect scale, improved significantly when wearing the robot. We also revealed that the salivary cortisol level, an indicator of stress, decreased significantly across all participants at the end of the experiment. In addition, participants’ fear of injections was significantly improved after participation in the experiment. These results suggest that the wearable soft robot may alleviate the human perception of pain and fear in during medical treatments, such as vaccinations.

## Introduction

Interpersonal touch can reduce human pain and fear^1–9^. This mechanism has been investigated by a broad range of disciplines from physiology, such as cutaneous receptors^10–22^, to psychology, such as emotion and well-being^23–32^. Moreover, the benefit of touch in nursing and therapy has been reported in clinical practice^33,34^.

Various haptic technologies and soft robotics have the potential to offer the benefits of interpersonal touch to humans, particularly in situations where human touch is unavailable (e.g., because of infection control^35–41^ or solitary situations^42–47^). In addition, incorporating the unique features of a social agent into these technologies may enable the replication of not only physical roles, such as haptic feedback and softness, but also social roles, such as empathy and bonding^48–54^. Indeed, a seal-shaped robot named PARO^55^ has been developed that is currently being used for therapeutic purposes^56–61^. A recent study reported that touching a PARO robot reduces the pain perception of adult participants^61^. However, although the body of the robot is covered in fur, and the participants can feel its softness by touch, the robot did not touch the participants, and its behavioral feedback was not manipulated; thus, the effect of interpersonal touch between the robot and human on the perception of pain and fear remains unclear.

To address this problem and determine whether interpersonal touch between a human and a social soft robot alleviates human pain and fear, we developed a hand-held soft robot that can be clenched by the wearer. The robot comprised small airbags that can be inflated to provide the wearer with a feeling of being clenched. This allowed us to test and compare three conditions of using the robot: [C1] no feedback (i.e., no robot movement), [C2] random feedback, and [C3] feedback in response to the wearer’s clench. In C1, the wearer could freely clench the robot, but the robot did not move. In C2, the robot inflated at random with no relevance to the wearer’s hand movement, and, in C3, the robot inflated in response to the wearer’s clench.

Experiments were conducted to test whether the robot alleviated the pain and fear of participants. We used a pain research protocol using thermal stimulation, which is well-established in the pain research field^61–63^. Participants wore the robot on their dominant hand, and thermal stimulation was applied to their non-dominant arm. We then measured participants’ subjective pain ratings using the pain assessment scale (PAS). In addition, we measured salivary oxytocin and cortisol levels from saliva samples collected from participants during the experiments. Oxytocin and cortisol levels have been used in pain research as an objective index to assess pain levels in humans^64–66^. We also measured participants’ fear of injections and psychological state before/after their participation in the experiments. Furthermore, given the possible future use of this robot in relieving pain and fear of individuals during medical procedures, such as vaccinations, we assessed whether the robot could alleviate participants’ fear of injections using the Injection Phobia Scale^67^.

## Results

In the experiments, participants carried out one of three conditions: [C1] no feedback, [C2] random feedback, and [C3] feedback in response to the wearer’s clench. Details of the experimental procedure, descriptions of the thermal stimulation, and the hand-held soft robot used in the experiments (Fig. 7) are provided in the Methods section.

The results reported in the following four sections comprise (1) PAS ratings, (2) salivary oxytocin and cortisol levels, (3) fear of injections, and (4) psychological state. Details of these four measurements are described in the Methods section.

### PAS ratings

**Figure 1:**
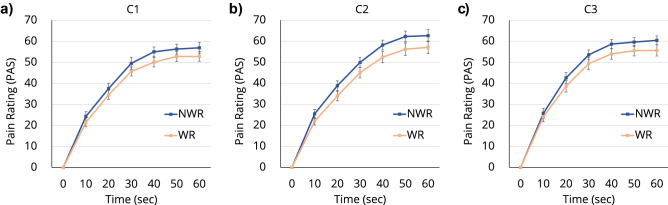
Reported PAS ratings by participants in the three conditions. Each participant rated their perceived pain level on the PAS every 10 s during a 60-s pain presentation.

**Figure 2:**
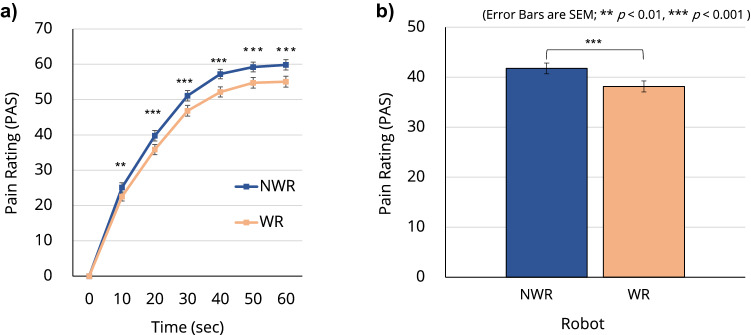
Post hoc comparisons of PAS ratings between NWR and WR. (**a**) Participants reported significantly lower pain ratings when they were WR than when they were NWR $(p< 0.01\, \text {at} 10\, \text {s and p} < 0.001 \text {at all the other time points})$. (**b**) A significant main effect of robot (NWR/WR) was also found.

Figure 1 shows the time transitions of the average PAS score of the participants in the three conditions. In each condition, there was a baseline measurement session where the participants did not wear the robot (NWR) but thermal stimulation was applied in the same manner as the experimental session in which they wore the robot (WR); the two corresponding lines are plotted in each of the three graphs in Fig. 1. A three-way analysis of variance (ANOVA), with a between-participants factor of condition for the three conditions and two within-participants factors of time and robot for the six 10-s intervals and the wearing of the robot, respectively, was performed to analyze the effects of the factors on participants’ subjective PAS scores. Although no significant interaction between condition and any other factor was found, there was a significant interaction between time and robot (F[6, 58] = 4.026, p = 0.002; Fig. 2a). There were also significant main effects of time $(\text {F}[6, 58] = 302.365, \text {p} < 0.001)$ and robot $(\text {F}[1, 63] = 23.737, \text {p} < 0.001)$. Post hoc comparisons revealed that participants reported significantly lower $(\text {p} < 0.01)$ pain ratings when they WR than when they did NWR (Fig. 2a,b; see Supplementary Table S1 for all related statistics).

### Salivary oxytocin and cortisol levels

**Figure 3:**
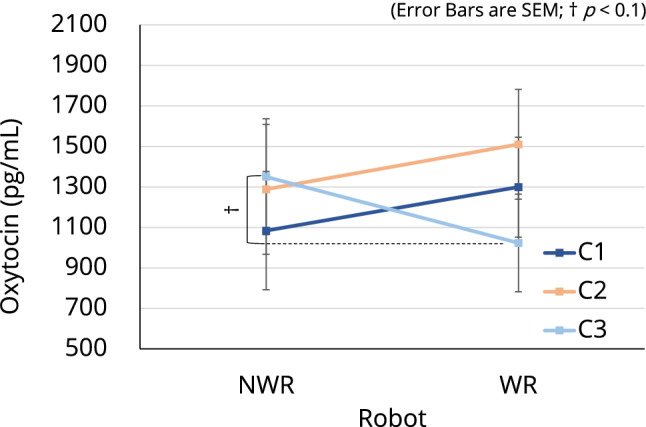
Salivary oxytocin levels of participants in the three conditions. A trend decrease (p = 0.051) in oxytocin level was observed when participants in condition C3 were WR compared with when they were NWR.

**Figure 4:**
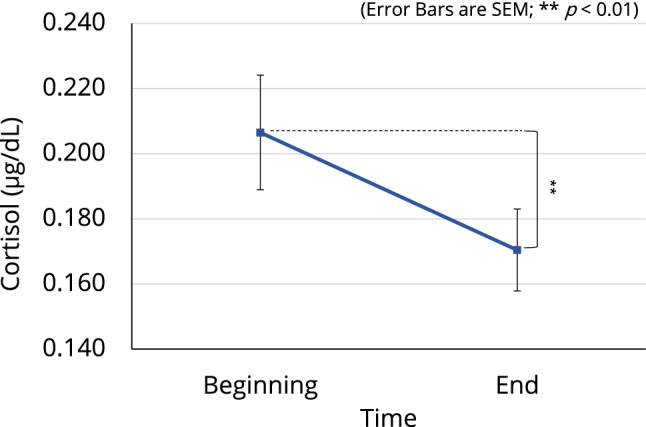
Overall change in cortisol level of participants from the beginning to the end of the experiment. A significant main effect of time was found $(p < 0.01)$. The figure illustrates a significant decrease $(p < 0.01)$ in cortisol level.

The three-way ANOVA with the three factors of condition, time, and robot revealed a significant interaction between condition and robot (F[2, 63] = 3.504, p = 0.036) for oxytocin level. However, no significant interaction nor the significant main effect was found for cortisol level. The post hoc comparison showed a trend decrease (p = 0.051) in oxytocin level when participants in condition C3 were WR compared with when they were NWR (Fig. 3; see Supplementary Table S2 for all related statistics). We also investigated the overall change in both oxytocin and cortisol levels from the beginning to the end of the experiment. Results showed a significant main effect of time for cortisol level (F[1, 63] = 9.275, p = 0.003). The post hoc comparison revealed that overall, participants’ cortisol level was significantly lower (p = 0.003) at the end of the experiment than at the beginning of the experiment (Fig. 4; see Supplementary Table S3 for all related statistics).

### Fear of injections

**Figure 5:**
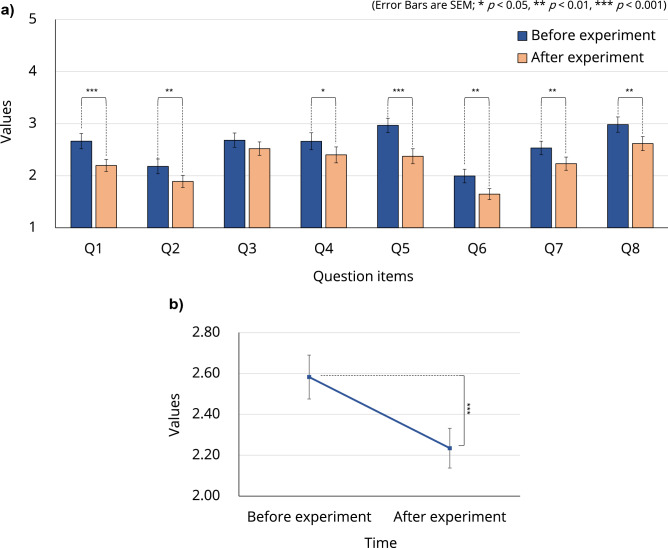
Participants’ fear of injections before and after the experiment. (**a**) Q1–Q8 asked participants’ fear for eight different scenarios related to injections (see Supplementary Table S4 for descriptions of these scenarios). (**b**) The average score for the eight questions before and after the experiment. The post hoc comparison revealed a significant decrease in the average score $(p < 0.001)$ after the experiment.

The Injection Phobia Scale comprises eight questions (Supplementary Table S4), which measure the subjective fear of participants across eight different scenarios related to injections. The ANOVA did not reveal a significant interaction; however, there were significant main effects $(\text {p} < 0.05)$ of time (before vs. after participation in the experiment) for all questions except Q3 (venepuncture) (Fig. 5a). Figure 5b shows the post hoc comparison result for the average score over the eight questions. There was a significant decrease $(\text {F}[1, 63] = 20.618, \text {p} < 0.001)$ in participants’ fear after participation in the experiment (see Supplementary Table S5 for all related statistics).

### Psychological state

**Figure 6:**
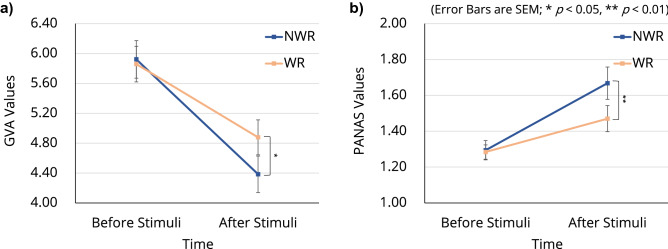
Psychological state of participants during the experiment. (**a**) Happiness state of GVA measured before and after each thermal stimulation. (**b**) Negative psychological state measured using the PANAS before and after each thermal stimulation.

The three-way ANOVA with the three factors of condition, time, and robot revealed significant interactions between time and robot for the happiness state of global vigor and affect (GVA; F[1, 63] = 4.128, p = 0.046) and negative psychological state assessed using the Positive and Negative Affect Scale $(\text {PANAS; F}[1, 63] = 7.186, \text {p} < 0.001)$. A significant main effect of time was found for both GVA $(\text {F}[1, 63] = 32.947, \text {p} < 0.001)$ and PANAS $(\text {F}[1, 63] = 20.318, \text {p} < 0.001)$ scores. A significant main effect of robot was found for negative psychological state assessed using the PANAS (F[1, 63] = 4.743, p = 0.033). The post hoc comparison revealed that participants’ happiness state after thermal stimulation was significantly higher (p = 0.017) when they were WR than when they were NWR (Fig. 6a). We also found that negative psychological state after thermal stimulation was significantly higher (p = 0.006) when participants were NWR than when they were WR (Fig. 6b; see Supplementary Table S6 for all related statistics).

## Discussion

We did not find significant interactions or a main effect of condition for PAS score, which suggested that (1) the three baseline sessions (where participants were NWR) were uniform for pain ratings across the three conditions, and (2) the three robot sessions (where participants were WR) were uniform for pain ratings across the three conditions (Fig. 1). The main effect of robot and the results of the post hoc comparisons suggested that WR reduces human pain perception, regardless of the control strategy used. PAS scores reduced by an average of 3.628 points (Supplementary Fig. S1).

Salivary oxytocin and cortisol levels are used in pain research as objective indicators of pain^64–66^. Saliva cortisol levels have been shown to increase with increases in stress levels^64^. However, changes in oxytocin levels are more complex and varied than those in cortisol levels. For example, it was reported that infant-caregiver interactions promote the secretion of oxytocin in both parties^68^, whereas oxytocin level has been shown to correlate with stress level^69^. A human–robot interaction (HRI) study that measured salivary oxytocin found that the oxytocin level decreased with the decrease in the stress level of participants^61^. However, studies on HRI are limited, and further investigations are needed to confirm the effects of HRI on oxytocin levels. In our study, we confirmed a downward trend in oxytocin level when participants in the C3 condition were WR (Fig. 3). We also found that these participants showed a reduction in pain perception as measured by the PAS (Fig. 2). Thus, our results for condition C3 suggested that haptic HRI induces a decrease in oxytocin level alongside a decrease in users’ pain perception. In addition, we observed a significant decrease in participants’ cortisol levels after participation in the experiment (Fig. 4), which suggested that the robot may alleviate pain and relax the user; this is consistent with the questionnaire results discussed below. This is the first study to report results for both oxytocin and cortisol levels simultaneously during HRI with respect to pain reduction.

Notably, the experience of WR contributed to the reduction in not only pain perception but also the fear of injections (Fig. 5). This was observed for questions Q2 (injection on the upper arm) and Q6 (immunization shot), which directly concern vaccinations, including those for coronavirus-19. Given that fear of injections is common^70^, the prospect of soft robotics solutions in reducing this fear may have considerable social impact. This is the first study to use the injection phobia scale while measuring pain during HRI. In addition, the analyses of the psychological state of participants using GVA and the PANAS revealed that participation in the experiment led to an overall improvement in their happiness state and negative psychological state (Fig. 6), which is consistent with the decrease in cortisol level discussed above.

This study has several limitations. First, the feedback control strategy of the robot requires further improvement to induce larger differences in effects between the C2 and C3 conditions. In our experiment, WR may have offered participants some degree of pain relief, regardless of the feedback control strategy of the robot. That is, participants may have perceived a degree of tactile sensation that was similar to that perceived while being clenched by the robot. Second, despite our best efforts in referring to previous studies^61,71–74^ for designing our experimental protocol, especially in regard to saliva measurements, further improvements to the timeline for measuring steady salivary oxytocin and cortisol levels are necessary. Because of the nature of hormonal activity and peripheral measurements, the interval and relaxation time between salivary measurements may have been prolonged. Third, sex differences in salivary oxytocin and cortisol levels and their association with pain perception were not considered. However, when sex was included as a between-participants factor, the female group showed a significant decrease in cortisol level following thermal stimulation when WR (Supplementary Fig. S2). Finally, we did not control for the robot factor (NWR/WR) to investigate participants’ fear of injections. Although the fear of injections reduced significantly after participating in the experiment, it remains unclear what element of the experiment induced this effect. Further studies are needed to address this question.

Despite these limitations, our study demonstrated the potential of social soft robotics and HRI in alleviating human pain/fear. A key element that would be of interest for future investigations is social factors, such as the agency^75^ of the robot and its social presence^76^. These can be explored using additional effective haptic feedback approaches. For example, the direction of gaze of the robot can be controlled to enable the robot to look toward or away from the wearer to determine whether gaze direction impacts pain perception. Furthermore, incorporating the latest augmented reality technology into the robot (e.g., by wearing a head-mounted display) will enable the introduction of further social HRI features, such as dialogue between the robot and the human. These potential research avenues will aid the development of solutions to pain/fear alleviation during medical procedures, such as vaccinations.

In regard to methodological/experimental aspects, electrical stimulation^77^, rather than thermal stimulation, may be used to explore the applicability of the robot to various treatment situations that involve pain as well as injection phobia. Furthermore, because we observed that interacting with the robot for a relatively short period in this study suppressed the reduction in the happiness state of GVA and the increase in the negative psychological state, future research could investigate the effect of robot-induced psychological state improvement on long-term stress.

## Methods

The main objective of this study was to examine whether human–robot interpersonal touch administered using a hand-held soft robot alleviates human pain and fear during a pain research protocol of thermal stimulation.

### Equipment

#### Inflatable hand-held soft robot

**Figure 7:**
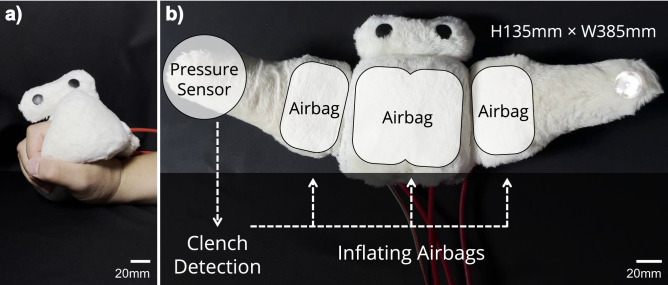
Inflatable hand-held soft robot. (**a**) The robot can be attached to the user’s hand. (**b**) Internal components were connected to an external pneumatic system with air pumps.

To generate human-robot interpersonal touch, we developed an inflatable hand-held soft robot (Fig. 7). Inside the robot were three airbags and a pressure sensor (Fig. 7b). The exterior of the robot was made of soft fur material. The robot was connected to an external pneumatic system composed of three air pumps and three solenoid valves that were independently controlled by a microcontroller (Arduino Nano V3.0, Arduino, Somerville, MA, USA). The wearer’s clench was detected by the pressure sensor. Depending on the control strategy, the three airbags were inflated to provide the wearer with a feeling of being clenched by the robot.

**Figure 8:**
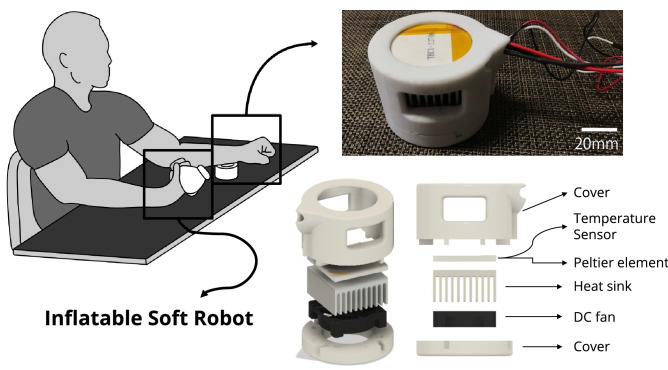
Thermal pain stimulator and experimental setup. Thermal stimuli were applied to the participant’s non-dominant arm using the thermal pain stimulator while the inflatable soft robot was placed on their dominant hand to perform haptic interaction with the robot (i.e., clenching the robot or being clenched by the robot).

#### Thermal pain stimulator

We employed a pain research protocol using thermal stimulation^62,63^. Thermal stimuli were generated and delivered using a thermal stimulator (Fig. 8). It had a 30-mm diameter contact probe, and its temperature was controlled using a Peltier element drive temperature controller (PLC-24V10A, Kurag Electronics, Tokyo, Japan) and a temperature sensor (103JT-025, SEMITEC Corporation, Tokyo, Japan). Following previous studies^61^, we applied thermal stimuli to the ventral side of the participant’s non-dominant arm (see Fig. 8 for the experimental setup) while the inflatable soft robot was placed on their dominant hand. The temperature of the thermal stimulation was either ${46}^\circ$ or ${47^\circ }$, which was determined in pilot experiments as corresponding to the Pain-60 level^62,63,78^.

### Measurements

The flow chart of all measurements is provided in Supplementary Fig. S3.

#### PAS rating

Subjective pain ratings were measured using the Allina Health PAS^79^. The original scale uses a scale from 0 to 10, where 0: no pain, 1–3: mild pain, 4–6: moderate pain, and 7–10: severe pain. According to previous pain research^62,63^, we used a scale that was modified by a factor of 10 (i.e., a scale ranging from 0 to 100). Participants verbally reported their pain rating whenever their rating changed.

#### Salivary oxytocin and cortisol levels

Oxytocin and cortisol levels of participants’ saliva samples were used as an objective pain indicator^64–66^. Saliva samples were collected in salivate tubes (Saliva Collection Aid and Cryovial, Salimetrcis LLC, State College, PA, USA) 2 min before and after the presentation of thermal stimulation. Saliva samples were collected four times during the experiment, and samples were stored at ${-\,20^\circ }$ until they were sent to Yanaihara Institute Inc. (Shizuoka, Japan) for assaying. Salivary oxytocin and cortisol measurements were performed using the oxytocin enzyme-linked immunosorbent assay (ELISA) kit (ADI-900-153A, Enzo Life Sciences, Farmingdale, NY, USA) and the cortisol (Saliva) enzyme immunoassay (EIA) kit (YK241, Yanaihara Research Institute, Shizuoka, Japan), respectively. The inter-assay coefficient of variance (CV) was $< 20.9\%$ and $< 5.9\%$, respectively, and the intra-assay CV was$< 13.3\%$ and $< 4.6\%$, respectively (calculated by each manufacturer). All assay procedures were performed according to manufacturer manuals. Participants were instructed in advance to take the following precautions for saliva collection:

1. Do not receive dental treatment within 24 h before salivation.
2. Do not take alcohol, caffeine, nicotine, or medicine within 12 h before salivation.
3. Do not eat foods high in sugar, acid or caffeine within 60 min before salivation.
4. Foods high in sugar, acid, and caffeine lower the pH of saliva and make it easier for bacteria to multiply.
5. Do not brush your teeth within 45 min before collecting saliva.
6. Rinse your mouth with water before measuring saliva, remove food debris, etc., and leave for at least 10 min.

#### Fear of injections

The eight-item short version of the Injection Phobia Scale^67^ was used to investigate whether interactions with the inflatable soft robot affected participants’ fear of injections (see Supplementary Table S4 for all question items). Each question item was rated on a five-point Likert-type scale ranging from 1 (no anxiety) to 5 (maximum anxiety). Fear of injections was measured as pre/post-questionnaires (Supplementary Fig. S3).

#### Psychological state

Participants’ psychological state was measured using the happiness state in GVA^80^ and negative state in the PANAS^81^. The happiness state was rated on a 10-point Likert-type scale ranging from 1 (very little) to 10 (very much). Negative psychological state was evaluated using 10 questions of the PANAS, which were rated on a five-point Likert-type scale ranging from 1 (very slightly or not at all) to 5 (extremely). Psychological state was measured when saliva collection was performed (Supplementary Fig. S3).

### Experimental setup

#### Participants

Seventy-two healthy adult participants were recruited from the University of Tsukuba and nearby junior colleges. None of the participants had acute or chronic pain, bruises on their arms, skin diseases, or mental illness, such as depression and communication disabilities. Participants were paid 2580 JPY as compensation for their participation. Data of six participants were excluded from all analyses because of non-hormone detection due to lack of saliva and outliers. The study protocol was approved by the Research Ethics Committee of the Faculty of Engineering, Information, and Systems at the University of Tsukuba (2020R376-2), and all participants provided written informed consent. All methods were performed in accordance with the relevant guidelines and regulations.

#### Procedures

The experiment consisted of two phases: phase 1 was a practice of the basic experimental procedure, and phase 2 was the main experimental session. The flow chart of these two phases is provided in Supplementary Fig. S3. All participants were informed of the precautions for saliva measurement in advance. The experiment was carried out in a quiet room, and the temperature was maintained at ${23^\circ }$. Participants were informed about the content of the experiment and completed the pre-questionnaires first. Then, participants completed the practice session to familiarize themselves with the procedure of the main experimental session in phase 2. To ensure safety and minimize the effect of sensitization^82^ (i.e., becoming hypersensitive to stimuli), thermal stimulation was presented alternately to two locations inside the forearm. The temperature of thermal stimulation used in the main experimental session was determined during this practice session by the following steps: (1) thermal stimulation at ${47^\circ }$ was applied for 1 min to the first location, followed by a 5-min rest, and then to the second location at the same temperature. (2) If the stimulation (${47^\circ }$) matched the participant’s Pain-60^62,63^ perception, the temperature was used for the main session, whereas if it exceeded the Pain-60 perception, the same procedure was repeated with a thermal stimulation of ${46^\circ }$, which was used for the main experimental session. Participants were randomly divided into three experimental condition (C1–C3) groups, which were matched for sex ratio. According to each condition, participants were instructed on how to interact with the inflatable soft robot during the practice session. Participants were also instructed to clench the robot whenever they felt pain. The participant carried out phase 2 twice (NWR and WR); the order was randomized to control for sequence effects (see box description in Supplementary Fig. S3). Participants wore noise-canceling headphones to reduce the influence of external sounds. After watching a relaxing video^83^ for 10 min to allow the effect from the practice session to return to baseline, participants completed the psychological state questionnaires, and saliva samples were collected. Participants then underwent a pain measurement procedure where thermal stimulation was administered for 1 min. After a 5-min interval, the same thermal stimulation procedure was repeated on the other forearm location. The mean value of the two pain measurements was used as the pain rating for each robot condition (NWR/WR). After the pain measurements, participants completed the psychological state questionnaires, and saliva samples were collected again. Participants then repeated the same procedure for the other robot wearing condition (i.e., WR or NWR) after watching a relaxing video. Finally, participants completed the post-questionnaires.

### Data analysis

The division of the 66 participants who provided valid data is shown in Supplementary Table S8. All dependent variables were tested for normality of distribution using Kolmogorov–Smirnov or Shapiro–Wilk analysis. ANOVA and Bonferroni post hoc analyses were conducted using the three factors of condition (C1, C2, and C3), time, and robot (NWR and WR); $\text {p} < 0.05$ was considered significant. All data were analyzed using IBM SPSS statistical software version 28 (IBM, Armonk, NY, USA).

## Supplementary Information

Supplementary Information.

## Data Availability

The data needed to evaluate the conclusions in the paper are present in the main manuscript or Supplementary Materials.
