# Supplementary material for: A wearable soft robot that can alleviate the pain and fear of the wearer
Source: Sci Rep. 2022 Oct 17;12:17003. doi: 10.1038/s41598-022-21183-7 (PMC9576738; doi:10.1038/s41598-022-21183-7)
Supplement: Supplementary file 1 — Supplementary Information. [file 41598_2022_21183_MOESM1_ESM.pdf]

Supplementary material

A Wearable Soft Robot that Can Alleviate the Pain and Fear of the Wearer

Youchan Yim, Yohei Noguchi, Fumihide Tanaka*

University of Tsukuba, Tsukuba, Japan.
*Corresponding author. Email: fumihide.tanaka@gmail.com

This PDF file includes:

- Fig. S1: PAS for rating pain during the experiment
- Fig. S2: Salivary cortisol levels of participants by sex
- Fig. S3: Flow chart of the experiment.
- Table S1: Results of pain ratings
- Table S2: Results of first saliva measurement
- Table S3: Results of the second saliva measurement
- Table S4: Injection Phobia Scale
- Table S5: Fear of Injections
- Table S6: Psychological State
- Table S7: Results of salivary cortisol measurements by sex
- Table S8: Information on the number and age of participants for each condition

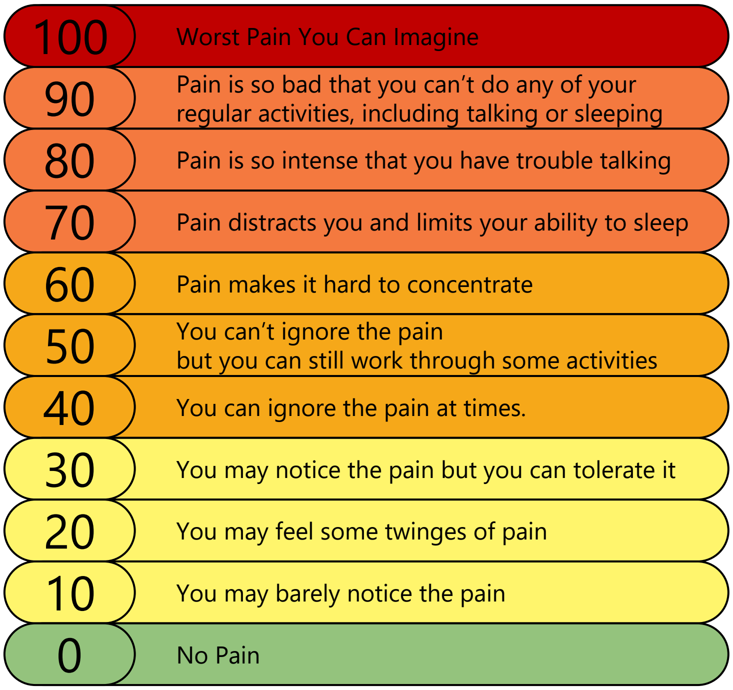

Fig. S1. PAS for rating pain during the experiment.

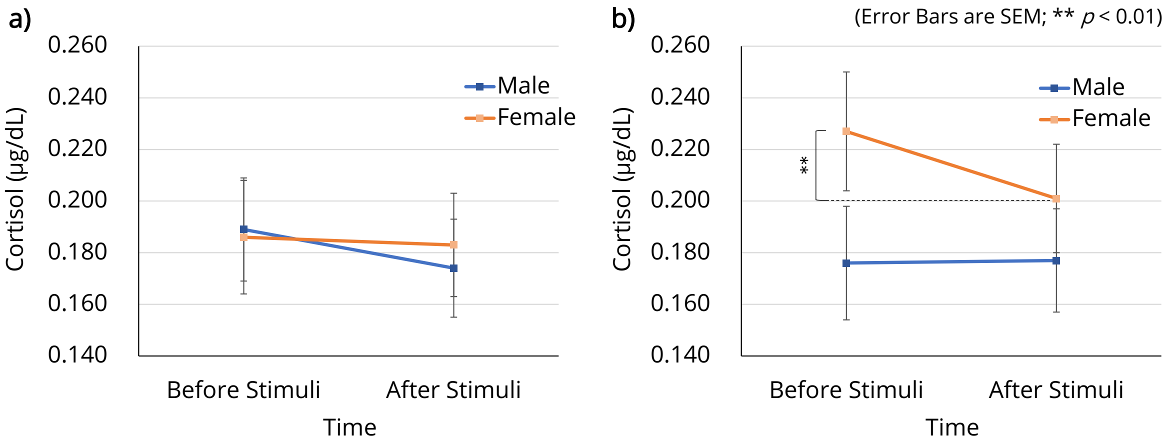

Fig. S2. Salivary cortisol levels of participants by sex. Analysis including the between-participants factor of sex showed a significant interaction ($p < 0.01$) between time, robot, and sex. (**a**) The interaction between time and sex when NWR was not significant ($p = 0.202$) for the analysis including the robot factor. (**b**) The interaction between time and sex when WR was significant ($p < 0.05$), and the cortisol level was significantly reduced after stimulation ($p < 0.01$), especially in females (see **Table S7** for all related statistics).

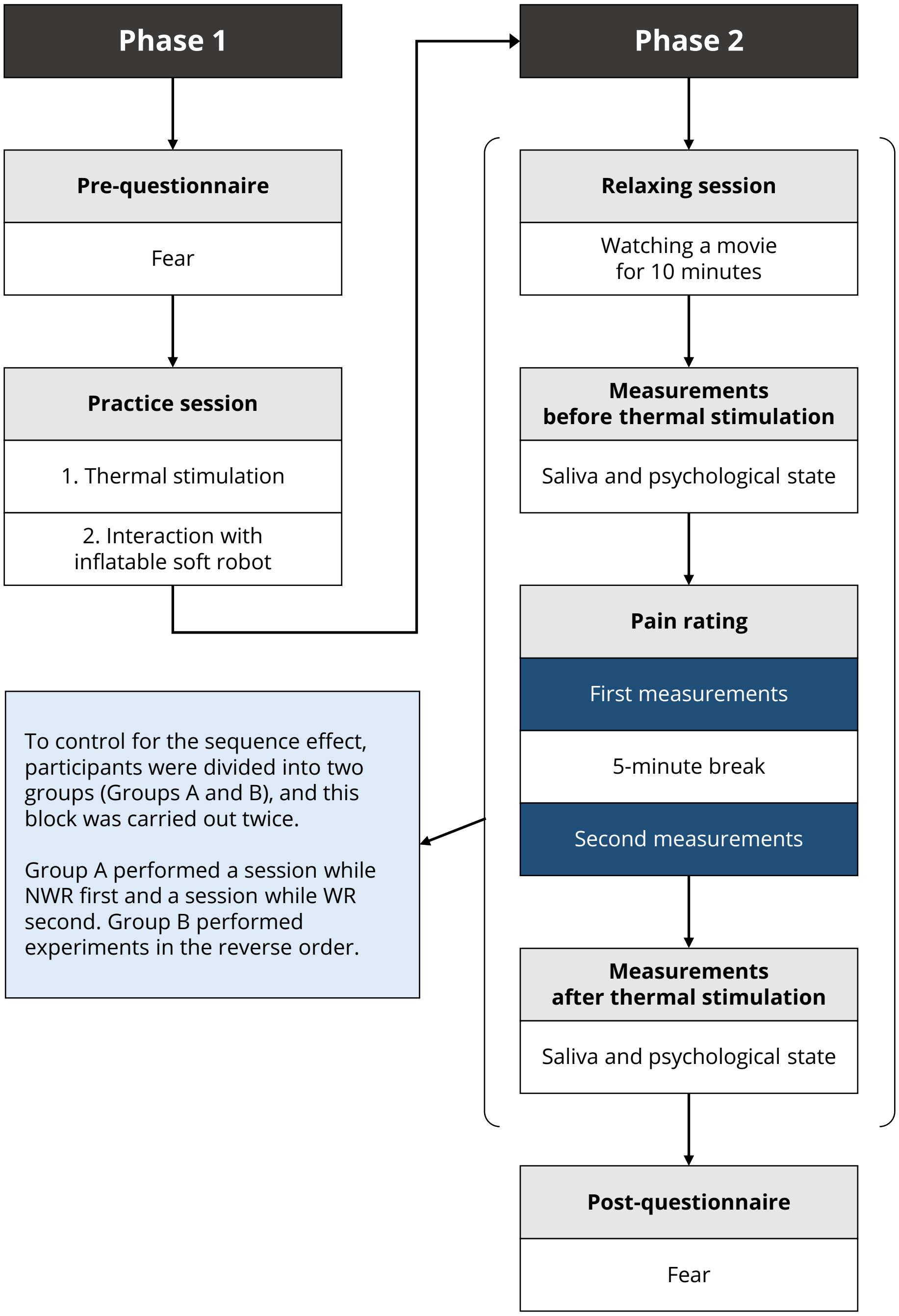

Fig. S3. Flow chart of the experiment.

Table S1. Results of pain ratings

**1. Results of Time × Robot × Condition three-way repeated measures ANOVA on pain ratings.**					
**Effect**	**F value**	**Hyp. Df**	**Error df**	**p-value**	η_p^2
Time	302.365	6	58	< 0.001	0.969
Robot	23.737	1	63	< 0.001	0.274
Condition	0.732	2	63	0.485	0.023
Time × Robot	4.026	6	58	0.002	0.294
Time × Condition	1.501	12	116	0.133	0.134
Robot × Condition	0.232	2	63	0.793	0.007
Time × Robot × Condition	0.665	12	116	0.782	0.064

**2. Results of bonferroni post hoc comparisons on pain ratings in Time × Robot interaction effect**					
**Effect**	**Time**	**Mean**		**Δ Mean**	**p-value**
		NWR	WR		
Time × Robot	T0	-	-	-	-
	T10	25.138	22.497	2.641	0.004
	T20	39.649	35.712	3.937	< 0.001
	T30	51.003	46.688	4.315	< 0.001
	T40	57.286	52.157	5.129	< 0.001
	T50	59.396	54.837	4.559	< 0.001
	T60	60.002	55.185	4.816	< 0.001

Table S2. Results of first saliva measurement

**1. Results of Time × Robot × Condition three-way repeated measures ANOVA on salivary oxytocin**					
**Effect**	**F value**	**Hyp. df**	**Error df**	**p-value**	η_p^2
Time	7.863	1	63	0.007	0.111
Robot	0.137	1	63	0.713	0.002
Condition	0.197	2	63	0.822	0.006
Time × Robot	0.199	1	63	0.657	0.003
Time × Condition	0.930	2	63	0.400	0.029
Robot × Condition	3.504	2	63	0.036	0.100
Time × Robot × Condition	2.100	2	63	0.131	0.062

**2. Results of Time × Robot × Condition three-way repeated measures ANOVA on salivary cortisol**					
**Effect**	**F value**	**Hyp. df**	**Error df**	**p-value**	η_p^2

Time	6.159	1	63	0.016	0.089
Robot	1.459	1	63	0.232	0.023
Condition	0.724	2	63	0.489	0.022
Time × Robot	0.262	1	63	0.610	0.004
Time × Condition	0.671	2	63	0.515	0.021
Robot × Condition	0.704	2	63	0.498	0.022
Time × Robot × Condition	2.108	2	63	0.130	0.063

3. Results of bonferroni post hoc comparisons on salivary oxytocin in Robot × Condition interaction effect

**Effect**	**Condition**	**Mean (pg/ml)**		**Δ Mean**	**p-value**
		**Robot**			
		NWR	WR		
Robot × Condition	C1	1083.811	1299.134	215.323	0.205
	C2	1288.028	1510.323	222.296	0.234
	C3	1350.257	1023.142	327.115	0.051

Table S3. Results of the second saliva measurement

1. Results of Time × Condition two-way repeated measures on salivary oxytocin

**Effect**	**F value**	**Hyp. df**	**Error df**	**p-value**	η_p^2
Time	0.443	1	63	0.508	0.007
Condition	0.329	2	63	0.721	0.010
Time × Condition	1.335	2	63	0.271	0.041

2. Results of Time × Condition two-way repeated measures on salivary cortisol

**Effect**	**F value**	**Hyp. df**	**Error df**	**p-value**	η_p^2
Time	9.275	1	63	0.003	0.128
Condition	0.561	2	63	0.574	0.017
Time × Condition	0.128	2	63	0.880	0.004

3. Results of bonferroni post hoc comparisons on salivary cortisol in Time main effect

**Effect**	**Saliva**	**Mean (μg/dL)**		**Δ Mean**	**p-value**
		**Time**			
		Beginning	End		
Time	Cortisol	0.207	0.170	0.036	0.003

Table S4. Injection Phobia Scale

No	Questionnaires
Q1	Giving a blood sample by having a finger pricked.
Q2	Having a shot in the upper arm.
Q3	Having a venipuncture (needle inserted into vein).
Q4	Getting an injection in the buttock.
Q5	Having one's ears pierced.
Q6	Getting a vaccination.
Q7	Getting an intravenous injection.
Q8	Having an anesthetic injection at the dentist.

Table S5. Fear of Injections

1. Results of Time × Condition two-way repeated measures ANOVA on each question and average score of Injection Phobia Scale						
**Question Number**	**Effect**	**F value**	**Hyp. df**	**Error df**	**p-value**	η_p^2
Q1	Time	20.400	1	63	< 0.001	0.245
	Condition	3.461	2	63	0.038	0.099
	Time × Condition	0.230	2	63	0.795	0.007
Q2	Time	9.955	1	63	0.002	0.136
	Condition	2.624	2	63	0.080	0.077
	Time × Condition	0.945	2	63	0.394	0.029
Q3	Time	2.412	1	63	0.125	0.037
	Condition	3.444	2	63	0.038	0.099
	Time × Condition	0.080	2	63	0.923	0.003
Q4	Time	4.973	1	63	0.029	0.073
	Condition	1.623	2	63	0.205	0.049
	Time × Condition	1.251	2	63	0.293	0.038
Q5	Time	25.413	1	63	< 0.001	0.287
	Condition	6.313	2	63	0.003	0.167
	Time × Condition	1.531	2	63	0.224	0.046
Q6	Time	11.533	1	63	0.001	0.155
	Condition	1.788	2	63	0.176	0.054
	Time × Condition	0.050	2	63	0.951	0.002
Q7	Time	8.783	1	63	0.004	0.122
	Condition	4.447	2	63	0.016	0.124
	Time × Condition	1.438	2	63	0.245	0.044

Q8	Time	8.922	1	63	0.004	0.124
	Condition	1.544	2	63	0.221	0.047
	Time × Condition	0.021	2	63	0.980	0.001
Average of all questions	Time	20.618	1	63	< 0.001	0.247
	Condition	4.690	2	63	0.013	0.130
	Time × Condition	0.589	2	63	0.558	0.018

2. Results of bonferroni post hoc comparisons on each question and average score of Injection Phobia Scale in Time main effect

**Question Number**	**Mean**		**Δ Mean**	**p-value**
	**Time**			
	Before experiment	After experiment		
Q1	2.664	2.197	0.468	< 0.001
Q2	2.179	1.889	0.291	0.002
Q3	2.682	2.519	0.163	0.125
Q4	2.662	2.401	0.261	0.029
Q5	2.967	2.374	0.593	< 0.001
Q6	1.994	1.647	0.347	0.001
Q7	2.533	2.232	0.301	0.004
Q8	2.982	2.617	0.364	0.004
Average of all questions	2.583	2.234	0.348	< 0.001

Table S6. Psychological State

1. Results of Time × Robot × Condition three-way repeated measures ANOVA on happiness state

**Effect**	**F value**	**Hyp. df**	**Error df**	**p-value**	η_p^2
Time	32.947	1	63	< 0.001	0.343
Robot	2.526	1	63	0.117	0.039
Condition	1.301	2	63	0.279	0.040
Time × Robot	4.128	1	63	0.046	0.061
Time × Condition	0.010	2	63	0.990	0.000
Robot × Condition	2.662	2	63	0.078	0.078
Time × Robot × Condition	0.295	2	63	0.745	0.009

**2. Results of Time × Robot × Condition three-way repeated measures ANOVA on negative state**					
**Effect**	**F value**	**Hyp. df**	**Error df**	**p-value**	**Partial Eta Squared**
Time	20.318	1	63	< 0.001	0.244
Robot	4.743	1	63	0.033	0.070
Condition	0.781	2	63	0.462	0.024
Time × Robot	7.186	1	63	< 0.001	0.102
Time × Condition	0.112	2	63	0.895	0.004
Robot × Condition	1.283	2	63	0.284	0.039
Time × Robot × Condition	2.189	2	63	0.121	0.065

**3. Results of bonferroni post hoc comparisons on happiness state in Time × Robot intera ction effect**					
**Effect**	**Time**	**Mean**		**Δ Mean**	**p-value**
		**Robot**			
		NWR	WR		
Time × Robot	Before Stimuli	5.922	5.859	0.063	0.734
	After Stimuli	4.384	4.878	0.494	0.017

**4. Results of bonferroni post hoc comparisons on negative state in Time × Robot interacti on effect**					
**Effect**	**Time**	**Mean**		**Δ Mean**	**p-value**
		**Robot**			
		NWR	WR		
Time × Robot	Before Stimuli	1.294	1.284	0.010	0.830
	After Stimuli	1.668	1.470	0.198	0.006

Table S7. Results of salivary cortisol measurements by sex

**1. Results of Time × Robot × Condition × Sex four-way repeated measures ANOVA on sali vary cortisol**					
**Effect**	**F value**	**Hyp. df**	**Error df**	**p-value**	η_p^2
Time	5.974	1	60	0.017	0.091
Robot	1.582	1	60	0.213	0.026
Condition	0.881	2	60	0.420	0.029
Sex	0.798	1	60	0.375	0.013

Time × Robot		0.543	1	60	0.464	0.009
Time × Condition		0.667	2	60	0.517	0.022
Time × Sex		0.589	1	60	0.446	0.010
Robot × Condition		0.725	2	60	0.488	0.024
Robot × Sex		2.867	1	60	0.096	0.046
Condition × Sex		0.681	2	60	0.510	0.022
Time × Robot × Condition		0.285	2	60	0.066	0.088
Time × Robot × Sex		8.019	1	60	0.006	0.118
Time × Condition × Sex		1.081	2	60	0.346	0.035
Robot × Condition × Sex		1.397	2	60	0.255	0.045
Time × Robot × Condition × Sex		1.073	2	60	0.348	0.035
**2. Results of Time × Sex two-way repeated measures ANOVA on salivary cortisol by Robot**						
**Robot**	**Effect**	**F value**	**Hyp. df**	**Error df**	**p-value**	η_p^2
NWR	Time	3.209	1	64	0.078	0.048
	Sex	0.012	1	64	0.912	0.000
	Time × Sex	1.663	1	64	0.202	0.025
WR	Time	3.858	1	64	0.054	0.057
	Sex	1.53	1	64	0.221	0.023
	Time × Sex	4.581	1	64	0.036	0.067
**3. Results of Time × Robot two-way repeated measures ANOVA on salivary cortisol by Sex**						
**Sex**	**Effect**	**F value**	**Hyp. df**	**Error df**	**p-value**	η_p^2
Male	Time	1.374	1	34	0.204	0.047
	Robot	0.24	1	34	0.627	0.007
	Time × Robot	3.17	1	34	0.084	0.085
Female	Time	4.751	1	30	0.037	0.137
	Robot	3.096	1	30	0.089	0.094
	Time × Robot	3.583	1	30	0.068	0.107
**4. Results of Robot × Sex two-way repeated measures ANOVA on salivary cortisol by Time**						
**Time**	**Effect**	**F value**	**Hyp. df**	**Error df**	**p-value**	η_p^2
Before	Robot	1.584	1	64	0.213	0.024

Stimuli	Sex	0.682	1	64	0.412	0.011
	Robot × Sex	5.627	1	64	0.021	0.081
After Stimuli	Robot	1.501	1	64	0.225	0.023
	Sex	0.374	1	64	0.543	0.006
	Robot × Sex	0.716	1	64	0.401	0.011

5. Results of bonferroni post hoc comparisons on salivary cortisol in Time × Sex interacti on effect

Robot	p-value of interaction effect	Sex	Mean (μg/dL) Time		Δ Mean	p-value
			Before Stimuli	After Stimuli		
NWR	0.202	Male	0.189	0.174	0.015	0.028
		Female	0.186	0.183	0.002	0.732
WR	0.036	Male	0.176	0.177	0.001	0.898
		Female	0.227	0.201	0.025	0.006

Table S8. Information on the number and age of participants for each condition

Condition	Order	Male	Female	Age (SD)	N	Total
C1	NWR first	6	6	21.75 (1.658)	12	**23**
	WR first	6	5	22.27 (2.149)	11	
C2	NWR first	6	4	21.20 (1.751)	10	**19**
	WR first	5	4	21.78 (1.787)	9	
C3	NWR first	5	7	23.58 (2.065)	12	**24**
	WR first	7	5	22.67 (1.614)	12	
**Total**		**35**	**31**	**22.26 (1.940)**	**66**	
